# Prognostic Factors for 10-Year Survival in Patients With Hepatocellular Cancer Receiving Liver Transplantation

**DOI:** 10.3389/fonc.2022.877107

**Published:** 2022-04-27

**Authors:** Quirino Lai, Andre Viveiros, Samuele Iesari, Alessandro Vitale, Gianluca Mennini, Simona Onali, Maria Hoppe-Lotichius, Marco Colasanti, Tommaso M. Manzia, Federico Mocchegiani, Gabriele Spoletini, Salvatore Agnes, Marco Vivarelli, Giuseppe Tisone, Giuseppe M. Ettorre, Jens Mittler, Emmanuel Tsochatzis, Massimo Rossi, Umberto Cillo, Benedikt Schaefer, Jan P. Lerut

**Affiliations:** ^1^General Surgery and Organ Transplantation Unit, Sapienza, Rome, Italy; ^2^Department of Medicine I, Innsbruck University, Innsbruck, Austria; ^3^Institut de Recherche Expérimental et Clinique (IREC), Université Catholique de Louvain, Brussels, Belgium; ^4^Department of Surgical, Oncological and Gastroenterological Sciences, Padua University, Padua, Italy; ^5^UCL Institute for Liver and Digestive Health and Royal Free Sheila Sherlock Liver Centre, Royal Free Hospital, London, United Kingdom; ^6^Klinik für Allgemein-, Viszeral- und Transplantationschirurgie, Mainz University, Mainz, Germany; ^7^Division of General Surgery and Liver Transplantation, San Camillo Hospital, Rome, Italy; ^8^Department of Transplant Surgery, PTV University, Rome, Italy; ^9^Unit of Hepatobiliary Surgery and Transplantation, Marche Polytechnic University, Ancona, Italy; ^10^Catholic University - Fondazione Policlinico Universitario Agostino Gemelli IRCCS, Rome, Italy

**Keywords:** recurrence, alpha-fetoprotein, radiological response, Milan criteria, expanded criteria

## Abstract

**Background:**

Long-term survival after liver transplantation (LT) for hepatocellular cancer (HCC) continues to increase along with the modification of inclusion criteria. This study aimed at identifying risk factors for 5- and 10-year overall and HCC-specific death after LT.

**Methods:**

A total of 1,854 HCC transplant recipients from 10 European centers during the period 1987–2015 were analyzed. The population was divided in three eras, defined by landmark changes in HCC transplantability indications. Multivariable logistic regression analyses were used to evaluate the significance of independent risk factors for survival.

**Results:**

Five- and 10-year overall survival (OS) rates were 68.1% and 54.4%, respectively. Two-hundred forty-two patients (13.1%) had HCC recurrence. Five- and 10-year recurrence rates were 16.2% and 20.3%. HCC-related deaths peaked at 2 years after LT (51.1% of all HCC-related deaths) and decreased to a high 30.8% in the interval of 6 to 10 years after LT. The risk factors for 10-year OS were macrovascular invasion (OR = 2.71; P = 0.001), poor grading (OR = 1.56; P = 0.001), HCV status (OR = 1.39; P = 0.001), diameter of the target lesion (OR = 1.09; P = 0.001), AFP slope (OR = 1.63; P = 0.006), and patient age (OR = 0.99; P = 0.01). The risk factor for 10-year HCC-related death were AFP slope (OR = 4.95; P < 0.0001), microvascular (OR = 2.13; P < 0.0001) and macrovascular invasion (OR = 2.32; P = 0.01), poor tumor grading (OR = 1.95; P = 0.001), total number of neo-adjuvant therapies (OR = 1.11; P = 0.001), diameter of the target lesion (OR = 1.11; P = 0.002), and patient age (OR = 0.97; P = 0.001). When analyzing survival rates in function of LT era, a progressive improvement of the results was observed, with patients transplanted during the period 2007–2015 showing 5- and 10-year death rates of 26.8% and 38.9% (vs. 1987–1996, P < 0.0001; vs. 1997–2006, P = 0.005).

**Conclusions:**

LT generates long-term overall and disease-free survival rates superior to all other oncologic treatments of HCC. The role of LT in the modern treatment of HCC becomes even more valued when the follow-up period reaches at least 10 years. The results of LT continue to improve even when prudently widening the inclusion criteria for transplantation. Despite the incidence of HCC recurrence is highest during the first 5 years post-transplant, one-third of them occur later, indicating the importance of a life-long follow-up of these patients.

## Introduction

Liver transplantation (LT) represents the gold-standard therapy to cure well-selected patients with hepatocellular cancer (HCC) (1). Before 1996, the absence of internationally recognized inclusion criteria explained the poor results of LT in patients with HCC (2). The introduction of the Milan criteria in clinical practice strongly modified the outcomes, resulting in 5-year survival rates similar to those obtained in non-HCC patients (3, 4). However, the rigorous adoption of these criteria significantly limits access to potentially successful treatment to a large number of patients, even slightly exceeding the selection criteria. Therefore, the transplant community widened in recent years the selection criteria for LT, thereby increasing the number of transplanted without impairing the expected results (5–7). Reporting of outcome is usually limited to 5-year survival rates. The impact of LT in the very long follow-up (i.e., ≥10 years) is still an unanswered question, especially when compared to other (curative) approaches such as liver resection (8).

In this light, it was hypothesized that LT should provide a beneficial 10-year survival impact. The study aimed at exploring the risk factors for 5- and 10-year death and HCC-specific death in a large international population of HCC liver patients.

## Methods

### Study Design

This is a retrospective international study carried out on prospectively maintained databases identifying adult (≥18 years) patients enlisted and transplanted with the primary diagnosis of HCC. This study followed the Strengthening the Reporting of Observational Studies in Epidemiology (STROBE) reporting guideline (9). The institutional review board of Azienda Ospedaliero-Universitaria Policlinico Umberto I (coordinating center) approved the study.

### Setting

Participants included 10 centers composing the EurHeCaLT Study Group. The centers participating in the study were as follows: Innsbruck University, Innsbruck, Austria (n = 296); Université Catholique de Louvain, Brussels, Belgium (n = 283); Padua University, Padua, Italy (n = 267); Sapienza University of Rome, Rome, Italy (n = 195); Royal Free Hospital, London, UK (n = 193); Mainz University, Mainz, Germany (n = 176); San Camillo Hospital, Rome, Italy (n = 142); PTV University Rome, Rome, Italy (n = 122); University of Marche, Ancona, Italy (n = 95); and Catholic University Rome, Rome, Italy (n = 85).

### Population

The investigated population included consecutive adult (≥18 years) patients enlisted and transplanted with the primary diagnosis of HCC during the period 1987–2015. Patients with HCC diagnosed only at pathological examination (incidental HCC), mixed hepatocellular-cholangiocellular cancer, and cholangiocellular cancer misdiagnosed as HCC were not included in the study.

### Variables and Data Collection

Collected patient-related data included the following: age and sex, cause of cirrhosis [hepatitis C virus (HCV), hepatitis B virus (HBV), alcohol, non-alcoholic steato-hepatitis (NASH), and other diseases], waiting time (WT) duration, model for end-stage liver disease (MELD), and period of LT (1987–1996, 1997–2006, and 2007–2015). Pre-LT available tumor-related data were morphologic HCC characteristics and alpha-fetoprotein (AFP) values evaluated at first referral and last pre-LT assessment, neo-adjuvant treatment(s), and subsequent modified Response Evaluation Criteria in Solid Tumors (mRECIST) status.

Tumor-related data obtained at pathological specimens were morphologic characteristics, multi-focality, bi-lobarity, poor grading, and micro- and macrovascular invasion. In all cases, morphologic HCC aspects referred to vital tumor tissue only.

### Definitions

Patient death was defined as any death caused by tumor- and non-tumor–related causes observed during the entire post-transplant follow-up. Patient death time was calculated as the time from LT to death after LT during the follow-up.

HCC-specific death was defined as a death directly caused by a tumor recurrence observed during the follow-up.

HCC recurrence was defined as any hepatic and/or extra-hepatic reappearance of the tumor at any time from the LT. Tumor recurrence time was calculated as the time from LT to detect tumor recurrence after LT during the follow-up. The last follow-up date was December 31, 2021.

The periods of LT were defined according to the introduction of some innovation in the field of transplant oncology: period 1987–1996 corresponding to the pre-Milan criteria era (liberal approach); (2) period 1997–2006 to the Milan criteria era; (3,4) and period 2007–2015 corresponding to the expanded criteria era (safe enlargement of inclusion criteria). In detail, the Up-to-seven criteria or the UCSF criteria were adopted in the different centers, with the exception of the Padua center, adopting the HCC-MELD score based on benefit principles (5–7).

### Statistical Analysis

Baseline characteristics of each data set were presented as medians and interquartile ranges (IQRs) for continuous variables and as numbers and percentages for discrete variables. Kruskal–Wallis test was adopted for comparing continuous variables. Chi-squared test was adopted for comparing dichotomous variables. Data missingness is detailed in **Supplementary Table 1**. In all the cases, covariates included in the analysis had missing data <10%. Missed data were handled with a single imputation method, and a median of nearby points was adopted (10).

Multivariable logistic regression analyses were used to evaluate the significance of independent risk factors for survival as independent prognostic factors for observed 5- and 10-year overall survival (OS) and for HCC-specific 5- and 10-year survival. The investigated variables were initially introduced using a “full model” approach, and then, the most relevant ones were selected using a backward Wald method with the intent to develop more parsimonious models. Odds ratios (ORs) and 95.0% confidence intervals (95.0% CIs) were reported.

Kaplan–Meier survival estimates were used to calculate survival curves. Log-rank test was used for comparing the survival distributions of different groups. A p-value <0.05 was considered statistically significant. Statistical analyses were conducted using SPSS 27.0 (SPSS Inc., Chicago, IL, USA).

## Results

### Patient and Tumor Characteristics

Patient and tumor characteristics are reported in **Table 1**. A total of 1,854 patients were enrolled for the present study. The median follow-up was 46.4 months (IQR: 16.4–90.0). A total of 751 (40.5%) and 256 (13.8%) patients overpassed the 5 and 10 years of follow-up, respectively.

**Table 1:** Patient demographic data and tumor features at first referral, last radiological assessment before LT, and pathological examination.

Variables	Median (IQR) or n (%)
Sex M/F	1,564/290 (84.4/15.6)
Age, years	57 (49–62)
Period of LT	
1987–1996	106 (5.7)
1997–2006	615 (33.2)
2007–2015	1,133 (61.1)
Waiting time, months	4 (2–9)
Underlying liver pathology*	
HCV	889 (48.0)
HBV	344 (18.6)
Alcohol	547 (29.5)
NASH	105 (5.7)
Other	132 (7.1)
MELD	12 (9–15)
Diameter of the target lesion, cm	
At first referral	2.5 (2.0–3.8)
Before LT	2.0 (1.0–3.0)
Number of nodules	
At first referral	1 (1–3)
Before LT	1 (1–3)
Milan criteria-out status	
At first referral	574 (31.0)
Before LT	404 (21.8)
AFP, ng/mL	
At first referral	10 (5–39)
Before LT	10 (5–33)
AFP slope ≥15 ng/ml/month	170 (9.2)
Type of response mRECIST after LRT	
Complete response	337 (18.2)
Partial response	535 (28.9)
Stable disease	219 (11.8)
Progressive disease	299 (16.1)
No LRT/no pre-LT evaluation after last LRT	464 (25.0)
Pre-LT LRT	1,524 (82.2)
Type of LRT**	
TACE	1,190 (64.2)
RFTA	367 (19.8)
PEI	321 (17.3)
Hepatic resection	173 (9.3)
TARE	26 (1.4)
SBRT	3 (0.2)
Pathological tumor features	
Diameter of the target lesion, cm	2.4 (1.5–3.5)
Number of nodules	2 (1–3)
Multifocality	976 (52.6)
Bilobar tumor	469 (25.3)
Poor grading	328 (17.7)
Microvascular invasion	394 (21.3)
Macrovascular invasion	56 (3.0)

* In some cases, same patients presented multiple pathologies. ** In some cases, same patients received multiple approaches.

IQR, interquartile ranges; n, number; M, male; F, female; LT, liver transplantation; HCV, hepatitis C virus; HBV, hepatitis B virus; NASH, non-alcoholic steato-hepatitis; MELD, model for end-stage liver disease; AFP, alpha-fetoprotein; mRECIST, modified Response Evaluation Criteria In Solid Tumors; LRT, loco-regional therapy; TACE, trans-arterial chemo-embolization; RFTA, radio-frequency termo-ablation; PEI, percutaneous ethanol injection; TARE, trans-arterial radio-embolization; SBRT, stereotactic body radiation therapy.

The median age of the patients was 57 years (IQR = 49–62), males (n = 1,564, 84.4%) largely outnumbered female patients. The main underlying liver disease was HCV, followed by alcoholic-related cirrhosis. The median MELD value was 12 (IQR = 9–15). The median duration of the waiting time was 4 months (IQR = 2–9).

Median diameter of the target lesion at time of LT was 2.0 cm, with a higher prevalence of single lesions. The median pre-LT AFP value was 10 ng/ml; 170 (9.2%) patients presented an AFP-slope >15 ng/ml/month during the waiting time. Neo-adjuvant treatment was applied in 82.2% of cases. Trans-arterial chemo-embolization (TACE) was the most commonly adopted loco-regional therapy (LRT), followed by radio-frequency ablation. Salvage LT after resection was carried out in 173 (9.3%) cases. A complete radiological tumor response was obtained in 337 (18.2%) of patients, and 299 (16.1%) patients had a progressive disease.

At pathological examination of the hepatectomy specimen, the median diameter of the target lesion was 2.4 cm, and the median number of lesions was 2. A poor tumor grading was observed in 328 (17.7%) cases. Micro- and macrovascular invasions were present in 394 (21.3%) and 56 (3.0%) patients, respectively.

### Patient Survival, HCC-Related Death, and Recurrence Estimates

**Figure 1:**
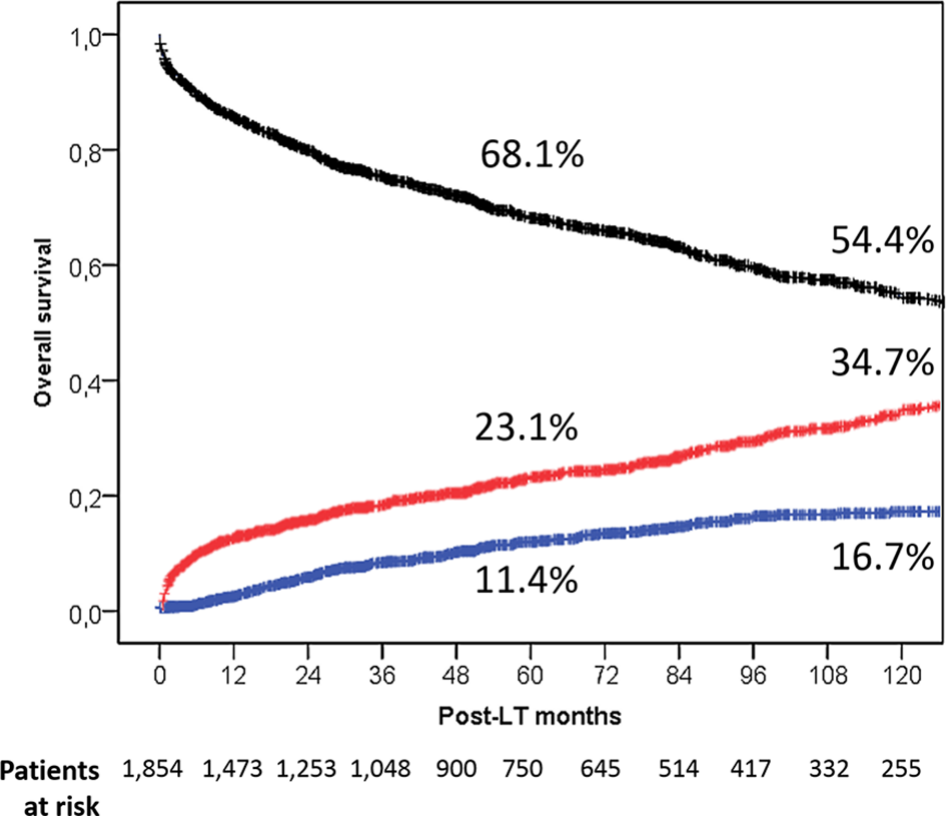
Overall patient survival rates in the entire population (black line). Death rates caused by tumor (blue line) and caused by other causes (red line) are also reported.

During the follow-up period, 651 of 1,854 (35.1%) liver patients died: 512 (27.6%) patients died within the first 5 years post-LT, 104 (5.6%) between 6 and 10 years, and 35 (1.9%) more than 10 years after LT. In **Table 2**, different measures of survival were reported. The 5- and 10-year Kaplan–Meier OS estimates were 68.1% and 54.4%, respectively (**Figure 1**).

**Table 2:** Different survivals rates in the analyzed population.

Survival rates (%)	1 year	2 years	3 years	4 years	5 years	6 years	7 years	8 years	9 years	10 years
Overall survival	85.6	79.9	75.3	72.0	68.1	66.0	63.2	59.5	57.5	54.4
HCC-related death	1.9	5.3	7.9	9.7	11.4	12.8	14.0	15.9	16.1	16.7
Non-HCC–related death	12.7	15.6	18.3	20.3	23.1	24.3	26.5	29.3	31.4	34.7
HCC recurrence	4.8	9.2	12.0	14.4	16.2	18.0	18.7	20.0	20.3	20.3

HCC, hepatocellular cancer.

A total of 180 (9.7%) and 471 (25.4%) deaths were HCC-related and no HCC-related, respectively. Five- and 10-year HCC-related and non-HCC–related death estimates were 11.4% and 16.7% vs. 23.1% and 37.4%, respectively (**Figure 1**).

**Figure 2:**
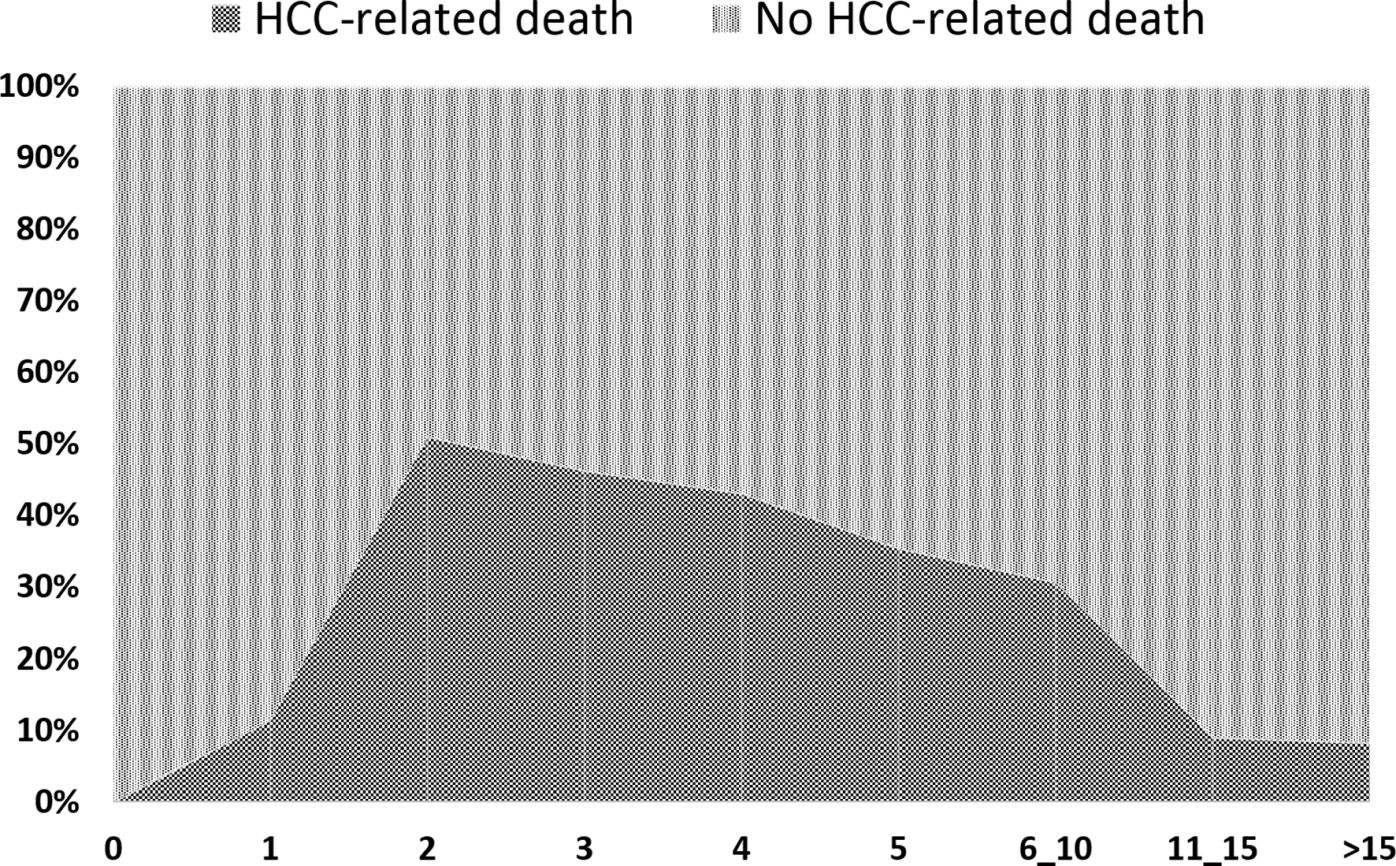
Causes of death expressed in percentages on the total number of cases at different time point of the follow-up.

In relation to the timeline of post-LT deaths, a fast increase of the tumor-related deaths was seen with a peak during the second post-LT year (51.1% of death causes). Later on, a slight decline was observed (third year = 46.4%; fourth year = 43.2; fifth year = 35.6%). The percentage of cancer-related deaths between 6 and 10 years post-LT was surprisingly high (30.8%). The risk of dying from an HCC-related cause lowered to 9.1% and 8.3%, respectively, during the post-LT periods of 11–15 and >15 years (**Figure 2**).

Two hundred forty-two (13.1%) recurrences were reported; 62 (3.4%) of these patients were still alive at the last follow-up. The 5- and 10-year Kaplan–Meier recurrence rates were 16.2% and 20.3%.

### Risk Factors for Overall Patient Death

Two separate multivariable logistic regression analyses were performed to explore the features connected with increased odds for the risk of 5- and 10-year death for any cause (**Table 3**). Observing the independent risk factors for 5-year death, macrovascular invasion showed the highest OR of 3.60 (P < 0.0001), followed by the diameter of the target lesion (OR = 1.12; P < 0.0001), poor grading (OR = 1.40; P = 0.01), AFP slope > 15 ng/ml/month (OR = 1.52; P = 0.02), and MELD score (OR = 1.02; P = 0.02).

**Table 3:** Multivariable logistic regression analysis for the risk of 5- and 10-year death after LT (backward Wald method).

Variables	Beta	SE	Wald	OR	95.0% CI		P-value
					Lower	Upper	
5-year death*							
Macrovascular invasion	1.28	0.31	17.25	3.60	1.97	6.58	<0.0001
Diameter target lesion cm	0.11	0.03	16.60	1.12	1.06	1.18	<0.0001
Poor grading (G3-4)	0.34	0.14	6.26	1.40	1.08	1.83	0.01
AFP slope >15 ng/ml/month	0.42	0.18	5.42	1.52	1.07	2.17	0.02
MELD	0.02	0.01	5.17	1.02	1.00	1.04	0.02
Constant	−1.71	0.16	112.60	0.18	–	–	<0.0001
10-year death**							
Poor grading (G3-4)	0.45	0.13	11.80	1.56	1.21	2.02	0.001
Diameter target lesion cm	0.09	0.03	11.28	1.09	1.04	1.14	0.001
Macrovascular invasion	0.997	0.31	10.38	2.71	1.48	4.97	0.001
HCV	0.33	0.10	10.28	1.39	1.14	1.69	0.001
AFP slope >15 ng/ml/month	0.49	0.18	7.68	1.63	1.15	2.30	0.006
Patient age	−0.01	0.01	5.98	0.99	0.98	0.997	0.01
Constant	−0.52	0.32	2.71	0.59	–	–	0.100

Hosmer–Lameshow test: *0.76; **0.49.

Variables initially tested in the model: patient age, sex, waiting list duration, HCV, HBV, alcohol, NASH, MELD, Milan criteria out at transplant, mRECIST complete response, mRECIST progressive disease, AFP value at transplant, AFP slope >15 ng/ml/month, diameter target lesion cm, number of nodules, multifocality, bilobarity, poor grading (G3-4), microvascular invasion, macrovascular invasion, pre-LT LRT, total number of LRT, salvage transplant after resection.

SE, standard error; OR, odds ratio; CI, confidence intervals; AFP, alpha-fetoprotein; MELD, model for end-stage liver disease; HCV, hepatitis C virus; HBV, hepatitis B virus; NASH, non-alcoholic steato-hepatitis; mRECIST, modified Response Evaluation Criteria In Solid Tumors; LT, liver transplantation; LRT, loco-regional therapy.

Recalculating the odds with a time horizon of 10 years, the following variables confirmed their negative prognostic impact: macrovascular invasion (OR = 2.71; P = 0.001), diameter of the target lesion (OR = 1.09; P = 0.001), poor grading (OR = 1.56; P = 0.001), and AFP slope (OR = 1.63; P = 0.006). In contrast, MELD score lost its relevance. HCV status (OR = 1.39; P = 0.001) and patient age (OR = 0.99; P = 0.01) reported statistically relevant odds in this long-term analysis.

### Risk Factors for HCCRelated Death

Two separate multivariable logistic regression analyses were utilized to explore the features connected with increased odds for the risk of 5- and 10-year HCC-related death (**Table 4**).

**Table 4:** Multivariable logistic regression analysis for the risk of 5- and 10-year HCC-related death after LT (backward Wald method).

Variables	Beta	SE	Wald	OR	95.0% CI		P-value
					Lower	Upper	
5-year HCC-related death*							
AFP slope >15 ng/ml/month	1.50	0.22	45.15	4.50	2.90	6.98	<0.0001
Microvascular invasion	0.71	0.22	10.60	2.02	1.32	3.10	0.001
Patient age	−0.03	0.01	9.73	0.97	0.95	0.99	0.002
Macrovascular invasion	1.04	0.35	8.96	2.82	1.43	5.57	0.003
Diameter target lesion cm	0.11	0.04	8.27	1.11	1.03	1.19	0.004
Poor grading (G3-4)	0.59	0.22	7.40	1.80	1.18	2.75	0.007
Number of nodules	0.06	0.02	6.81	1.07	1.02	1.12	0.009
MELD score	−0.05	0.02	4.43	0.95	0.91	0.997	0.04
Constant	−1.47	0.63	5.46	0.23	–	–	0.02
10-year HCC-related death**							
AFP slope >15 ng/ml/month	1.60	0.21	56.95	4.95	3.27	7.49	<0.0001
Microvascular invasion	0.76	0.20	14.88	2.13	1.45	3.12	<0.0001
Poor grading (G3-4)	0.67	0.20	11.39	1.95	1.32	2.88	0.001
Patient age	−0.03	0.009	10.93	0.97	0.95	0.99	0.001
Total number of LRT	0.10	0.03	10.19	1.11	1.04	1.18	0.001
Diameter target lesion cm	0.11	0.04	9.48	1.11	1.04	1.19	0.002
Macrovascular invasion	0.84	0.34	6.14	2.32	1.19	4.52	0.01
Constant	−1.98	0.52	14.67	0.14	–	–	<0.0001

Hosmer–Lameshow test: *0.13; **0.39.

Variables initially tested in the model: patient age, sex, waiting list duration, HCV, HBV, alcohol, NASH, MELD, Milan criteria out at transplant, mRECIST complete response, mRECIST progressive disease, AFP value at transplant, AFP slope >15 ng/ml/month, diameter target lesion cm, number of nodules, multifocality, bilobarity, poor grading (G3-4), microvascular invasion, macrovascular invasion, pre-LT LRT, total number of LRT, salvage transplant after resection.

SE, standard error; OR, odds ratio; CI, confidence intervals; HCC, hepatocellular cancer; AFP, alpha-fetoprotein; MELD, model for end-stage liver disease; LRT, loco-regional therapy; HCV, hepatitis C virus; HBV, hepatitis B virus; NASH, non-alcoholic steato-hepatitis; mRECIST, modified Response Evaluation Criteria In Solid Tumors; LT, liver transplantation.

Again, similar variables were observable in the two models. As for the risk of 5-year HCC-specific death, AFP slope had the highest OR of 4.50 (P < 0.0001), followed by microvascular invasion (OR = 2.02; P = 0.001), macrovascular invasion (OR = 2.82; P = 0.003), diameter of the target lesion (OR = 1.11; P = 0.004), poor grading (OR = 1.80; P = 0.007), and number of nodules (OR = 1.07; P = 0.009). Patient age (OR = 0.97; P = 0.002) and MELD score (OR = 0.95; P = 0.04) were protective for the risk of HCC-specific death.

When the time horizon was set at to 10 years, the relevant role of AFP slope was confirmed (OR = 4.95; P < 0.0001), followed by microvascular (OR = 2.13; P < 0.0001) and macrovascular invasion (OR = 2.32; P = 0.01), poor tumor grading (OR = 1.95; P = 0.001), total number of neo-adjuvant therapies (OR = 1.11; P = 0.001), and diameter of the target lesion (OR = 1.11; P = 0.002). Again, patient age was a protective factor (OR = 0.97; P = 0.001).

### Correlation Between Death and Period of Transplant

Relevant differences existed among the different periods in terms of patient and tumor characteristics and clinical management, as reported in **Table 5**. In light of these aspects, a sub-analysis was done focused on the different risk factors for 10-year HCC-related death in the three different periods (**Table 6**). In detail, the slope of AFP was always the most relevant risk factors in all the different periods. The number of LRT emerged as a detrimental factor only in the last two periods, in which the pre-LT management with multiple LRT has raised as a routine approach in HCC transplant candidates.

**Table 5:** Patient demographic data and tumor features at first referral and last radiological assessment before LT in the three different periods.

Variables	1987–1996 (n = 106, 5.7%)	1997–2006 (n = 615, 33.2%)	2007–2015 (n= 1,133, 61.1%)	P-value
	Median (IQR) or n (%)			
Sex M/F	84/22 (79.2/20.8)	517/98 (84.1/15.9)	963/170 (85.0/15.0)	0.29
Age, years	51 (41–58)	55 (40–61)	58 (52–63)	<0.0001
Waiting time, months	1 (0–3)	5 (2–10)	4 (2–9)	<0.0001
Underlying liver pathology*				
HCV	45 (42.5)	295 (48.0)	549 (48.5)	0.50
HBV	31 (29.2)	121 (19.7)	192 (16.9)	0.005
Alcohol	15 (14.2)	167 (27.2)	365 (32.3)	<0.0001
NASH	1 (0.9)	30 (4.9)	74 (6.5)	0.04
Other	22 (20.8)	34 (5.5)	76 (6.7)	<0.0001
MELD	12 (12–12)	12 (10–15)	12 (9–15)	0.03
Diameter of the target lesion, cm				
At first referral	3.0 (2.5–5.0)	2.5 (2.0–3.7)	2.5 (1.9–3.7)	<0.0001
Before LT	1 (1–3)	1 (1–2)	1 (1–3)	0.08
Number of nodules				
At first referral	3.0 (2.0–5.2)	2.0 (1.2–3.0)	1.8 (0.8–2.8)	<0.0001
Before LT	1 (1–3)	1 (1–3)	1 (1–3)	0.13
Milan criteria-out status				
At first referral	46 (43.4)	175 (28.5)	353 (31.2)	0.009
Before LT	48 (45.3)	114 (18.5)	242 (21.4)	<0.0001
AFP, ng/mL				
At first referral	13 (6–195)	13 (5–52)	10 (5–30)	<0.0001
Before LT	31 (88–385)	10 (5–41)	8 (4–24)	<0.0001
AFP slope ≥15 ng/ml/month	37 (34.9)	45 (7.3)	88 (7.8)	<0.0001
Type of response mRECIST after LRT				
Complete response	1 (0.9)	99 (16.1)	237 (20.9)	<0.0001
Partial response	17 (16.0)	180 (29.3)	338 (29.8)	0.01
Stable disease	4 (3.8)	103 (16.7)	112 (9.9)	<0.0001
Progressive disease	6 (5.7)	72 (11.7)	221 (19.5)	<0.0001
No LRT/no pre-LT evaluation after last LRT	79 (74.5)	161 (26.2)	224 (19.8)	<0.0001
Pre-LT LRT	33 (31.1)	511 (83.1)	980 (86.5)	<0.0001
Type of LRT**				
TACE	23 (21.7)	408 (66.3)	759 (67.0)	<0.0001
RFTA	0 (-)	61 (9.9)	306 (27.0)	<0.0001
PEI	8 (7.5)	116 (18.9)	197 (17.4)	0.02
Hepatic resection	4 (3.8)	46 (7.5)	123 (10.9)	0.009
TARE	0 (-)	0 (-)	26 (2.3)	<0.0001
SBRT	0 (-)	0 (-)	0 (-)	0.38

* In some cases, same patients presented multiple pathologies. ** In some cases, same patients received multiple approaches.

IQR, interquartile ranges; n, number; M, male; F, female; LT, liver transplantation; HCV, hepatitis C virus; HBV, hepatitis B virus; NASH, non-alcoholic steato-hepatitis; MELD, model for end-stage liver disease; AFP, alpha-fetoprotein; mRECIST, modified Response Evaluation Criteria In Solid Tumors; LRT, loco-regional therapy; TACE, trans-arterial chemo-embolization; RFTA, radio-frequency termo-ablation; PEI, percutaneous ethanol injection; TARE, trans-arterial radio-embolization; SBRT, stereotactic body radiation therapy.

**Table 6:** Multivariable logistic regression analysis for the risk of 10-year HCC-related death after LT (backward Wald method) in the three different periods.

Variables	Beta	SE	Wald	OR	95.0% CI		P-value
					Lower	Upper	
1987–1996*							
AFP slope >15 ng/ml/month	3.84	0.85	20.66	46.64	8.90	244.54	<0.0001
Microvascular invasion	1.58	0.58	7.33	4.83	1.54	15.13	0.007
Milan criteria out	−1.63	0.85	3.67	0.20	.04	1.04	0.055
Constant	−2.50	0.51	23.87	0.08	–	–	<0.0001
1997–2006**							
AFP slope >15 ng/mlmonth	1.62	0.36	20.11	5.07	2.50	10.32	<0.0001
Poor grading	1.18	0.29	16.38	3.26	1.84	5.77	<0.0001
Number of nodules	0.12	0.05	6.73	1.13	1.03	1.24	0.009
Total number of LRT	0.13	0.05	6.27	1.14	1.03	1.25	0.01
Microvascular invasion	0.58	0.30	3.88	1.79	1.00	3.18	0.049
Constant	**–**3.10	0.25	155.56	0.05	–	–	<0.0001
2007–2015***							
AFP slope >15 ng/ml/month	1.26	0.35	13.30	3.54	1.79	6.97	<0.0001
HBV	1.25	0.39	10.43	3.50	1.64	7.50	0.001
Diameter target lesion	0.16	0.05	9.13	1.18	1.06	1.31	0.003
Microvascular invasion	0.84	0.31	7.61	2.33	1.28	4.24	0.006
Macrovascular invasion	1.35	0.53	6.62	3.87	1.38	10.86	0.01
Total number of LRT	0.12	0.05	4.84	1.12	1.01	1.24	0.03
Milan criteria out	0.70	0.32	4.72	2.01	1.07	3.78	0.03
HCV	0.69	0.35	3.99	2.00	1.01	3.93	0.046
Constant	−5.18	0.43	146.36	0.01	–	–	<0.0001

Hosmer–Lameshow test: *0.78; **0.32; ***0.16.

Variables initially tested in the model: patient age, sex, waiting list duration, HCV, HBV, alcohol, NASH, MELD, Milan criteria out at transplant, mRECIST complete response, mRECIST progressive disease, AFP value at transplant, AFP slope >15 ng/ml/month, diameter target lesion cm, number of nodules, multifocality, bilobarity, poor grading (G3-4), microvascular invasion, macrovascular invasion, pre-LT LRT, total number of LRT, salvage transplant after resection.

SE, standard error; OR, odds ratio; CI, confidence intervals; HCC, hepatocellular cancer; AFP, alpha-fetoprotein; MELD, model for end-stage liver disease; LRT, loco-regional therapy; HCV, hepatitis C virus; HBV, hepatitis B virus; NASH, non-alcoholic steato-hepatitis; mRECIST, modified Response Evaluation Criteria In Solid Tumors; LT, liver transplantation.

**Figure 3:**
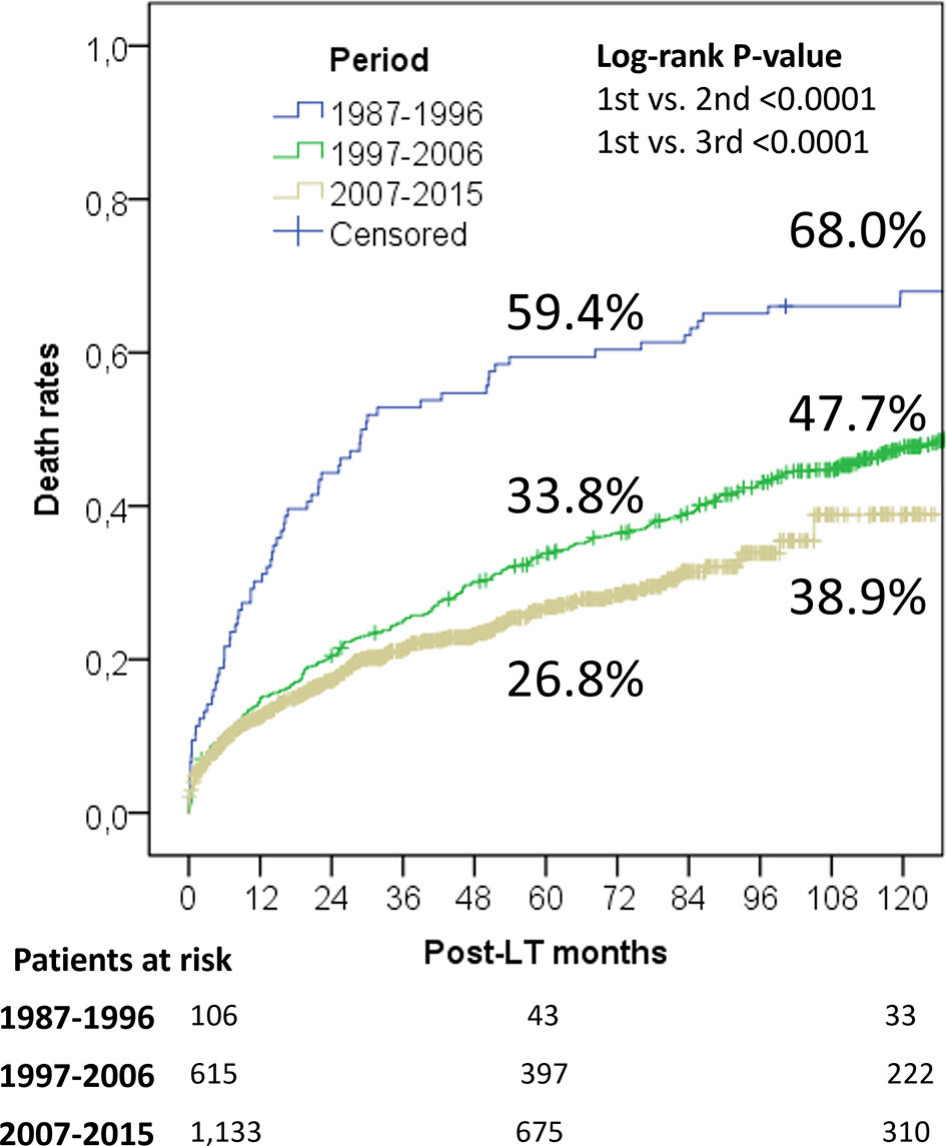
Evolution of death rates (all the causes) in the different periods of transplantation.

When analyzing survival rates in function of LT era, a progressive improvement of the results was observed (**Figure 3**). Patients transplanted during the 1987–1996 “liberal era”, characterized by the absence of any recognized inclusion criterion, had exceedingly high 5- and 10-year overall death rates of 59.4% and 68.0%. As expected, the results improved significantly during the 1997–2006 “Milan criteria era”, with 5- and 10-year death rates declining to of 33.8% and 47.7%. Log-rank test showed a statistically relevant difference between these two eras (P < 0.0001). Last, the results further improved during the 2007–2015 “safe criteria enlargement era”, with 5- and 10-year death rates of 26.8% and 38.9%. During this latter period, Milan criteria were progressively expanded by introducing San Francisco and Up-to-seven criteria. Interestingly, log-rank analysis survival rates were significantly improved when compared to those obtained during the first (P < 0.0001) and second era (P = 0.005).

Five- and 10-year HCC-related death rates were 35.6% and 41.7%, 12.6% and 18.0%, and 8.0% and 11.3% during the periods 1987–1996, 1997–2006, and 2007–2015, respectively. It was interesting to note that the latter period showed better results despite a slight enlargement of the criteria was adopted during this period respect to the previous one (P = 0.005).

## Discussion

In the present study, the 5- and 10-year survival rates of 68.1% and 54.4% observed in a large European cohort containing 1,854 patients with HCC compared favorably with the widely accepted lower limit for 5-year patient survival after LT of 50% (11).. These results are in line with findings reported in large international databases such as the European Liver Transplant Registry (ELTR), which reported, in a cohort of 18,349 HCC liver patients, 5- and 10-year survival rates of 66% and 51%, respectively (12).

Compared to all other therapeutic modalities, the long-term superiority of LT does not disserve sufficient attention within the medical community, although well known since long time (13).

A recent Chinese study including 1,255 patients with HCC compared the 10-year survival outcomes from three different first-line treatments, namely, radiofrequency ablation, liver resection, and transplantation. LT was clearly superior in terms of 10-year survival, even after adjustment for confounders and balancing of the compared cohorts using inverse probability weighting (8). A meta-analysis comparing LT and resection as the treatment options in small HCC meeting the Milan criteria reported that the 5-year OS rates were similar, whereas the 10-year rates were significantly higher in patients who underwent LT than resection (50.0 vs. 29.8%; P < 0.001) (14).

These findings were also confirmed when the concept of “transplant benefit” was investigated. Exploring the data of 1,028 HCC cirrhotic patients coming from one Eastern and two Western surgical units, the 10-year scenario increased drastically the transplant benefit in all subgroups of resectable patients, and LT became an effective therapy for all patients without microvascular invasion independent of tumor extension and for oligo-nodular HCC with microvascular invasion meeting the conventional Milan and San Francisco criteria (15).

The present study confirms that a combination of morphological and biological tumor variables is linked to risk of death and HCC recurrence. A large US experience including 3,276 patients validated the Risk Estimation of Tumor Recurrence After Transplant (RETREAT) score, consisting of AFP value at LT, microvascular invasion, and the sum of the largest viable tumor and number of tumors in the total hepatectomy specimen (16). Interestingly, all these variables were statistically relevant risk factors for 10-year HCC-related death in our series.

Moreover, we explored the AFP dynamics during the waiting time instead of looking at the last available value before LT. Several studies assigned a relevant role to the AFP slope as a predictor for recurrence and death (17–19).

Macrovascular invasion is another relevant variable that has been recently explored in large international series. A retrospective study analyzing 45 patients with macrovascular patients before LT reported a very high risk of recurrence especially if the AFP value at LT was >10 ng/ml (5-year disease-free survival rates 27.8 vs. 71.8%; P = 0.008) (20). A ELTR study (n = 9,324) reported that vascular invasion overruled as prognostic indicator all criteria based on size and number of nodules; 5-year OS rates reached 39.6%, 58.8%, and 73.2% in patients with macrovascular invasion, microvascular invasion, or absent invasion (21). All these experiences are in line with our findings. Both micro- and macrovascular invasion at pathological examination of the hepatectomy specimen correlated with poor tumor-related survival. The growing role of advanced locoregional therapies like the radio-embolization is showing promising results in terms of efficacious downstaging of macrovascular invasion using “superdownstaging” protocols (22).

In relation to the total number of neo-adjuvant treatments, several studies explored the negative effect of repeated therapies as a surrogate of a more aggressive tumor behavior. A large US experience including 789 Milan criteria-out HCC patients reported a detrimental effect of LRT in patients failing to be successfully downstaged when compared to directly transplanted patients (HCC recurrence: 34.1% vs. 26.1%; p < 0.001) (23). A European experience based on the analysis of 1,083 Milan criteria-in patients reported that up to three LRTs are beneficial for success in intention-to-treat LT patients, but, if patients need more LRT, this benefit is lost (24). Our series confirmed that the risk for long-term tumor-related death was increased in patients requiring more LRT, supporting the hypothesis that the need for more LRT is equivalent to higher tumor aggressiveness.

Our study explored the impact of risk factors available at the time of LT only, whereas other relevant aspects such as the role of immunosuppressive treatment were not. Despite there is some recent evidence that immunosuppression either as maintenance or anti-rejection treatment may play a role as another risk factor for HCC recurrence after transplantation, it was not explored because intending to investigate only variables that are available at the time of LT (25, 26). The investigation of variables obtainable after LT in fact introduces an “immortal bias” into the analysis. This potential risk was avoided excluding all the post-LT variables.

Our analysis confirmed the negative role of HCV infection on the long-term survival. During the studied time period, early viral allograft reinfection was universal. Nowadays, direct-acting antiviral agents have almost eliminated this risk for death. Therefore, it has to be foreseen that HCV infection will lose its role as a relevant risk factor for long-term death (27).

In the future, the prevalence of NASH will become the main reason to LT in patients with HCC, and this underlying disease will replace very soon HCV as a risk factor for delayed death after LT (28). In our series, the impact of NASH appears to be relatively limited, but its raising role is clearly reported observing the growing number of cases observed in the different LT periods.

Interestingly, in our series many patients (71 of 242; 29.3%) recurred very late (>5 years). Unfortunately, it was impossible to analyze more in detail if these recurrences were “real” ones or *de novo* HCCs in the transplanted graft (29, 30). The very late detection of HCC in this series suggests that one should be very cautious when declaring a patient cured from HCC if no recurrence has been diagnosed within 5 years after LT and also underlines the importance of a long-life oncologic follow-up (31).

Another interesting aspect to highlight is the fact that a high number of patients with HCC with recurrence were still alive at the time of last follow-up. This finding further underlines the role of screening protocols, which represent the only way to early diagnose and, whenever possible, aggressively treat the recurrence (32). In this setting, the beneficial role of the new systemic therapies is unexplored. However, the potential ability of these drugs to prevent or to manage mid- and long-term recurrence requires further attention (33, 34).

The study presents some limitations. First, this is a retrospective analysis, but the great majority of studies focusing on transplant oncology derive from retrospective cohorts. Second, this study is based on a large European experience with an extended enrolment period (1987–2015). Such a long-time span leads to several potential biases linked to a modified and improved tumor and patient management. The enrolment of patients transplanted during the earlier periods was necessary to document long-term oncologic results and patient survivals post-LT. To mitigate potential biases, the variable “era of LT” was introduced in the mathematical models, and several sub-analyses focused on the different periods were performed. The multicenter nature of the study is likely to add another bias due to some differences in relation to HCC policies in the different centers. The enrollment of large patient numbers should mitigate a “center-related” effect, minimizing the potential impairment caused by different waiting times, neo-adjuvant strategies, and center volumes. Moreover, the composition of this European collaborative group was based on a similar interest and approach toward patients with HCC selected for a potential liver transplantation (LT). Last, the inclusion criteria of patients with HCC for LT changed during the study period, moving from a “liberal” approach *via* the exclusive use of the Milan criteria to the more recent use of the expanded criteria. Therefore, the variable “LT era” was introduced in the mathematical models and a LT period–oriented analysis was also performed to look at the effect of changes in the treatment of HCC in potential liver patients.

In conclusion, LT generates long-term overall and disease-free survival rates which are superior to all other oncologic treatments of HCC. The role of LT in the modern treatment of HCC becomes even more valued when the follow-up period reaches at least 10 years. The results of LT continue to improve even when prudently widening the inclusion criteria for transplantation. Despite the fact that the incidence of HCC recurrence is highest during the first 5 years post-transplant, one-third of them occur later on, indicating the importance of a life-long follow-up of these patients.

## Data Availability Statement

The raw data supporting the conclusions of this article will be made available by the authors, without undue reservation.

## Ethics Statement

The studies involving human participants were reviewed and approved by AOU Policlinico Umberto I Rome. Written informed consent for participation was not required for this study in accordance with the national legislation and the institutional requirements.

## Author Contributions

QL and JL contributed to conception and design of the study. QL, AnV, SI, AlV, GM, SO, MH-L, MC, TM, FM, and GS contributed to acquisition of data. QL analyzed and interpreted the data. QL and JL drafted the article. SA, MV, GT, GM, JM, ET, MR, UC, BS, and JL critically revised the manuscript. All authors contributed to the article and approved the submitted version.

## Conflict of Interest

The authors declare that the research was conducted in the absence of any commercial or financial relationships that could be construed as a potential conflict of interest.

## Publisher’s Note

All claims expressed in this article are solely those of the authors and do not necessarily represent those of their affiliated organizations, or those of the publisher, the editors and the reviewers. Any product that may be evaluated in this article, or claim that may be made by its manufacturer, is not guaranteed or endorsed by the publisher.

## Abbreviations

AFP, alpha-fetoprotein; CI, confidence interval; ELTR, European Liver Transplant Registry; HBV, hepatitis B virus; HCC, hepatocellular cancer; HCV, hepatitis C virus; IQR, interquartile range; LRT, loco-regional therapy; LT, liver transplantation; MELD, model for end-stage liver disease; mRECIST, modified Response Evaluation Criteria in Solid Tumors; NASH, non-alcoholic steato-hepatitis; OR, odds ratio; OS, overall survival; RETREAT, Risk Estimation of Tumor Recurrence After Transplant; STROBE, Strengthening the Reporting of Observational Studies in Epidemiology; TACE, trans-arterial chemo-embolization; WT, waiting time.
