# Supplementary material for: Prognostic Factors for 10-Year Survival in Patients With Hepatocellular Cancer Receiving Liver Transplantation
Source: Front Oncol. 2022 Apr 27;12:877107. doi: 10.3389/fonc.2022.877107 (PMC9093683; doi:10.3389/fonc.2022.877107)
Supplement: Supplementary file 1 [file Table_1.docx]

**Additive data**

**Additive Table 1.** Missing data.

| **Variables** | **Cases available (%)** | **Missing cases (%)** |
| --- | --- | --- |
| Sex | 1,854 (100.0) | 0 (-) |
| Age | 1,854 (100.0) | 0 (-) |
| Period of LT | 1,854 (100.0) | 0 (-) |
| Waiting time months | 1,854 (100.0) | 0 (-) |
| HCV | 1,854 (100.0) | 0 (-) |
| HBV | 1,854 (100.0) | 0 (-) |
| Alcohol | 1,854 (100.0) | 0 (-) |
| NASH | 1,854 (100.0) | 0 (-) |
| Other | 1,854 (100.0) | 0 (-) |
| MELD | 1,670 (90.1) | 184 (9.9) |
| Diameter of the target lesion (first referral) | 1,739 (93.8) | 115 (6.2) |
| Number of nodules (first referral) | 1,744 (94.1) | 110 (5.9) |
| Diameter of the target lesion (before LT) | 1,764 (95.1) | 90 (4.9) |
| Number of nodules (before LT) | 1,767 (95.3) | 87 (4.7) |
| AFP (first referral) | 1,744 (94.1) | 110 (5.9) |
| AFP (before LT) | 1,827 (98.5) | 27 (1.5) |
| Any radiological response after LRT | 1,720 (92.8) | 134 (7.2) |
| Pre-LT LRT | 1,854 (100.0) | 0 (-) |
| TACE | 1,854 (100.0) | 0 (-) |
| RFTA | 1,854 (100.0) | 0 (-) |
| PEI | 1,854 (100.0) | 0 (-) |
| Hepatic resection | 1,854 (100.0) | 0 (-) |
| TARE | 1,854 (100.0) | 0 (-) |
| SBRT | 1,854 (100.0) | 0 (-) |
| Diameter of the target lesion (pathology) | 1,854 (100.0) | 0 (-) |
| Number of nodules (pathology) | 1,854 (100.0) | 0 (-) |
| Multifocality (pathology) | 1,854 (100.0) | 0 (-) |
| Bilobar tumor (pathology) | 1,751 (94.4) | 103 (5.6) |
| Poor grading (pathology) | 1,854 (100.0) | 0 (-) |
| Microvascular invasion (pathology) | 1,854 (100.0) | 0 (-) |
| Macrovascular invasion (pathology) | 1,852 (99.9) | 2 (0.1) |
| **Abbreviations:** LT, liver transplantation; HCV, hepatitis C virus; HBV, hepatitis B virus; NASH, non-alcoholic steato-hepatitis; MELD, model for end-stage liver disease; AFP, alpha-fetoprotein; LRT, loco-regional therapy; TACE, trans-arterial chemo-embolization; RFTA, radio-frequency thermo-ablation; PEI, percutaneous ethanol injection; TARE, trans-arterial radio-embolization; SBRT, stereotactic body radiation therapy. | | |
